# Case Report: A case of HNF1B mutation patient with first presentation of diabetic ketosis

**DOI:** 10.3389/fendo.2022.917819

**Published:** 2022-08-05

**Authors:** Shenghui Ge, Mengge Yang, Wenfeng Gong, Wenzhe Chen, Jianjun Dong, Lin Liao

**Affiliations:** ^1^ Department of Endocrinology and Metabology, The First Affiliated Hospital of Shandong First Medical University & Shandong Provincial Qianfoshan Hospital, Ji-nan, China; ^2^ Cheeloo College of Medicine, Shandong University, Department of Endocrinology and Metabology, Shandong Provincial Qianfoshan Hospital, Shandong Key Laboratory of Rheumatic Disease and Translational medicine, Shandong Institute of Nephrology, Ji-nan, China; ^3^ Division of Endocrinology, Department of Internal Medicine, Qilu Hospital of Shandong University, Ji-nan, China

**Keywords:** MODY5, HNF1B, case, mutation, diagnosis, renal cysts and diabetes syndrome

## Abstract

**Background:**

Maturity-onset diabetes of the young 5 (MODY5), a rare diabetes syndrome of young adults, is associated with variants in hepatocyte nuclear factor 1B (*HNF1B*) gene.

**Case Presentation:**

We reported a case of MODY5, which presented with diabetic ketosis, multiple renal cysts, and hypokalemia. In this case, the *HNF1B* score was estimated as 13 and a heterozygous variant of *HNF1B* in exon 4 (c.826C>T, p.Arg276*) was identified through Sanger sequencing.

**Conclusions:**

Multiple renal cysts and youth-onset diabetes are common manifestations in patients with *HNF1B* mutations, and insufficient insulin secretion may be a potential cause of diabetic ketosis in MODY5.

## Background

Diabetes, as an early adverse factor in many other diseases, has attracted widespread attention, and there is growing evidence that diabetes is a heterogeneous disease affected by genetic and environmental influences. With the maturity of genetic testing technology, single-gene defect diabetes has been continuously discovered, especially maturity-onset diabetes of the young (MODY). Currently, at least 14 genes have been confirmed to be involved in the pathogenesis of MODY, including *GCK*, *HNF1A*, *HNF1B*, *HNF4A*, *PDX1*, *NEUROD-1*, *KLF-11*, *CEL*, *PAX4*, *INS*, *BLK*, *ABCC8*, *KCNJ11*, and *APPL1* (1). Maturity-onset diabetes of the young 5 (MODY5) is expected to account for less than 5% of MODY, which is mainly related to *HNF1B* gene variation (2). However, there is a large heterogeneity in its clinical manifestations. Therefore, it is necessary for us to expand on the clinical phenotype of MODY5 so that clinicians can better identify this disease. In this report, we describe a MODY5 patient with diabetic ketosis, which provides a basis for the supplement of the MODY5 phenotype.

## Case presentation

**Figure 1:**
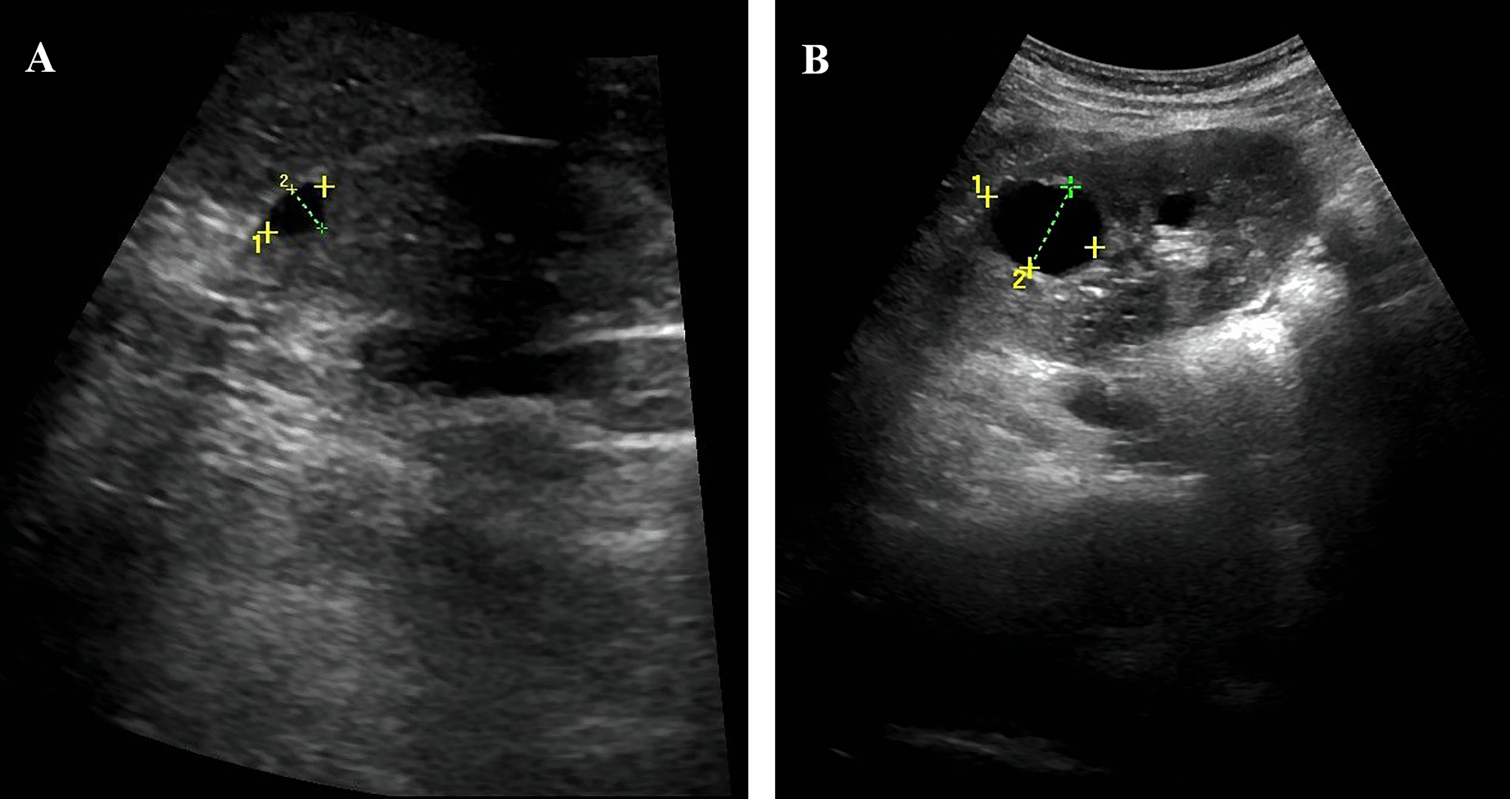
Abdominal ultrasound showed the patient’s kidneys. **(A)** The patient has multiple cysts in the right kidney, the largest cyst (9*6mm). **(B)** The patient has multiple cysts in the left kidney, the largest cyst (25*19mm).

A 26-year-old Chinese woman was admitted to our hospital, with symptoms of thirst, easy hunger, and polyuria for half a year. She had no nausea, vomiting, diarrhea, abdominal pain, or blurred vision. The patient was 156 cm in height, 37.6 kg in weight, and 15.5 kg/m^2^ in body mass index (BMI). Physical examination revealed no abnormalities except rapid pulse rate and malnutrition. Laboratory investigations revealed a random blood glucose (RBG) level of 28.9 mmol/L and glycated hemoglobin (HbA1c) value of 17.40%. The erythrocyte sedimentation rate (ESR) level was 10 mm/h and c-reactive protein was less than 3.11 mg/L, both within the normal range. Urine ketone bodies were positive and urine sugar was strongly positive. Therefore, the patient was diagnosed with diabetic ketosis and treated with insulin at a dose of 0.48 U/kg*d as a tentative pharmacotherapy. The patient’s hyperglycemia gradually improved and urine ketones were negative. After that, we performed the relevant examination for the patient (**Tables 1** and **2**). The results showed that her fasting glucose (FBG) level was 9.08 mmol/L and serum C-peptide and insulin levels were 0.31 nmol/L and 2.03 uIU/mL, and 2-hour postprandial blood glucose level was 9.75 mmol/L and 2-hour postprandial serum C-peptide and 2-hour postprandial insulin levels were 0.26 nmol/L and 1.40 uIU/mL. The patient’s insulin resistance index HOMA-IR was estimated as 0.82. Serum creatinine and esti mated glomerularfiltrationrate (eGFR) were 57.00 umol/L and 129.23, respectively. Serum potassium level was low and serum magnesium level was at the lower limit of normal. As shown in **Figure 1**, ultra-sonographic examination of the abdomen revealed multiple cysts in both kidneys, the echogenicity of the liver parenchyma was enlarged, and no obvious abnormality was detected in other organs. To determine the pathogenesis of her diabetes, further tests were performed (**Table 1**). Anti-GAD, anti-IAA and anti-ICA antibodies, unique to type 1 diabetes, were all negative. The patient’s insulin secretion was insufficient, so insulin subcutaneous pump was used and the dose was maintained at 0.38 U/kg*d.

**Table 1:** Laboratory examinations at the time of admission.

	Subject	Value	Normal range
Blood routine	Leukocytes (×10^9^/L)	6.11	3.50-9.50
	Erythrocyte (×10^12^/L)	4.50	3.80-5.10
	Platelets (×10^9^/L)	198.00	125.00-350.00
	Hemoglobin (g/L)	135.00	115.00-150.00
Liver function	ALT (IU/l)	7.40	7.00-40.00
	AST (IU/l)	11.70	13.00-35.00
	γGTP (IU/l)	10.00	7.00-45.00
	ALP (IU/l)	48.00	35.00-100.00
	Total protein (g/dl)	66.90	65.00-85.00
	Albumin (g/dl)	40.00	40.00-55.00
Renal function	eGFR	129.23	
	Uric acid (umol/L)	334.00	142.80-339.20
	Creatinine (umol/L)	57.00	45.00-84.00
Urine routine	Erythrocyte	(-)	(-)
	Leukocytes	(-)	(-)
	Urine protein	(-)	(-)
	Urine glucose	(++++)	(-)
	Ketone bodies	(++)	(-)
Electrolyte	Na (mmol/L)	141.00	137.00-147.00
	K (mmol/L)	3.13	3.50-5.30
	Cl (mmol/L)	102.40	99.00-110.00
	Ca (mmol/L)	2.21	2.09-2.54
	Pi (mmol/L)	0.75	0.87-1.45
	Mg (mmol/L)	0.76	0.70-1.10
Blood lipid	Total cholesterol (mmol/L)	4.37	3.17-6.17
	Triglyceride (mmol/L)	1.19	0.41-1.77
	HDL-cholesterol (mmol/L)	1.20	0.98-1.94
	LDL-cholesterol (mmol/L)	2.30	1.84-3.76
Inflammatory	C-reactive protein (mg/dl)	< 3.11	0.00-3.48
	ESR (mm/h)	10.00	0.00-20.00
	PCT (ng/mL)	0.04	0.00-0.05
Thyroid related indicators	FT3 (pmol/L)	3.25	3.10-6.80
	FT4 (pmol/L)	23.07	12.00-22.00
	TSH (uIU/mL)	1.60	0.27-4.20
	TPOAB (IU/mL)	18.80	0.00-34.00
	TgAB (IU/mL)	26.29	0.00-115.00
Autoantibodies in diabetes	GADA (IU/mL)	5.97	0.51-30.00
	ICA	(-)	(-)
	IAA (IU/mL)	3.67	0.41-20.00
Glycemic parameters	HbA1c (%)	17.40	4.00-5.60%

ALT, alanine aminotransferase; AST, aspartate aminotransferase; γGTP, γ-glutamyl transferase; ALP, a lkaline phosphatase; eGFR, esti mated glomerularfiltrationrate; ESR, erythrocyte sedimentation rate; PCT, procalcitonin; FT3, free triiodothyronine; FT4, free thyroxine; TSH, thyroid stimulating hormone; TPOAB, thyroidperoxidase antibodies; TgAB, thyroglobulin antibody; GADA, glutamic acid decarboxylase antibody; IAA, insulin autoantibody; ICA, islet cell antibody; HbA1c, glycated hemoglobin.

**Table 2:** Fasting and 2-hour postprandial metabolic indicators.

Subject	0h	2h	Normal range
Plasma glucose (mmol/L)	9.08	9.75	3.89-6.11
Serum C-peptide (nmol/L)	0.31	0.26	0.37-1.47
Serum insulin (uIU/mL)	2.03	1.40	2.60-24.90

**Figure 2:**
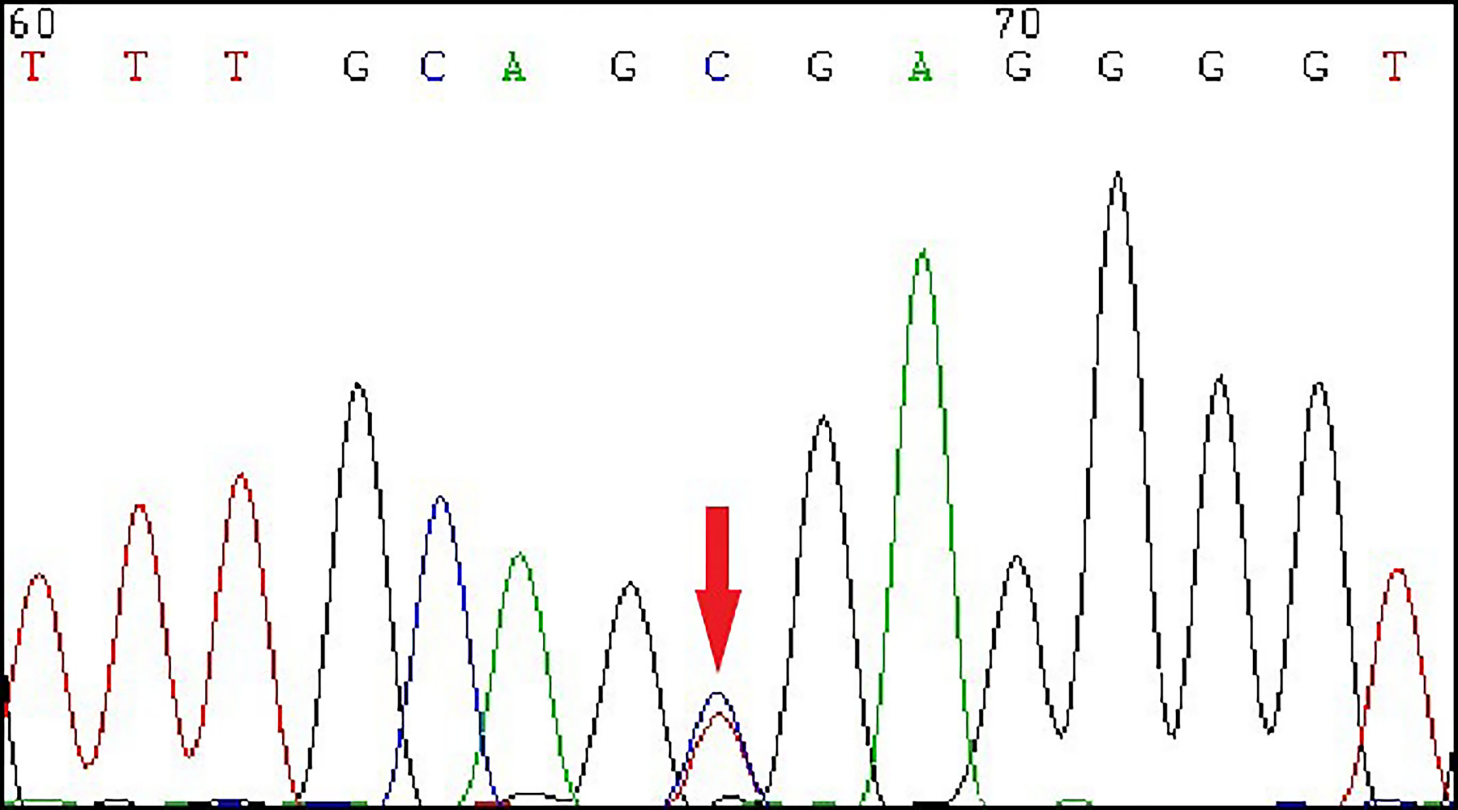
The Sanger sequencing showed a heterozygous mutation of *HNF1B* gene in exon 4 (c.826C>T, p.Arg276*) of the patient.

Due to the presence of young-onset diabetes, low BMI, multiple renal cysts in both kidneys, insulin secretion dysfunction, and negative diabetes-related antibodies, we suspected the patient to be MODY5 and the *HNF1B* score was estimated as 13 (**Table S1**) (3). To further establish definitive diagnosis, genomic DNA was isolated from peripheral blood of the patient, and DNA sequence analysis of the *HNF1B* gene revealed a heterozygous point mutation (c.826C>T) in 4 exon, leading to a nonsense change of amino acids 276, which changed the amino acid position 276 of the encoded protein from Arg to stop codon (**Figure 2**). Therefore, the result indicated that our patient was MODY5. This mutation has been reported previously (**Table 3**).

**Table 3:** Cases of *HNF1B* mutation (c.826C>T, p.Arg276*) in literature.

	Case1	Case2	Case3	Case4	Case5
Reference	(4)	(5)	(6)	(7)	This case
Country	Japan	Japan	Brazil	China	China
Gender	Male	Male	Female	Female	Female
Age (years)	20	13	14	24	26
BMI (kg/m^2^)	17.3	23.2	21.9	17.9	15.5
Family history of diabetes	Y	–	–	Y	–
Autoantibodies in diabetes	N	–	–	N	N
Ketone body	–	–	–	–	Y
Multiple renal cyst	Y	Y	N	Y	Y
Pancreas	–	–	–	Hypoplasia	Normal
Hypokalemia	N	N	–	–	Y
Hypomagnesemia	–	–	–	Y	N
Treatment of diabetes	insulin	insulin	insulin	insulin	insulin

Age, age at diagnosis of diabetes; BMI, body mass index; Y, yes; N, no.

After her discharge, insulin subcutaneous pump was used with the basal dosage 2.0 units/d and bolus 4.0, 5.8 and 2.5 units before breakfast, lunch and dinner. She tested her blood glucose frequently. Her levels of fasting glucose were around 4 to 6 mmol/L, post prandial 7 to 10 mmol/L, and the recent HbA1c was 5.1%.

## Discussion

MODY5 is a rare disease characterized by multiple cysts of both kidneys, pancreatic dysplasia, hypomagnesemia, and diabetes in young adults, usually caused by mutations in *HNF1B*. While this was first reported by Horikawa in 1997, now several 100 mutations have been identified (8, 9). The *HNF1B* is expressed in the pancreas, liver, and kidney, and plays an important role in the development of multiple tissues (10–12). MODY5 can be classified into 2 entities according to the genetic defect type: patients with a gene deletion and patients with a gene mutation. Patients with *HNF1B* mutations had a poorer renal prognosis than those with a gene deletion (13).

In our patient, heterozygous point mutation (c.826C>T, p.Arg276*) occurred in exon 4 and diabetic ketosis was the first manifestation. This was the first report of diabetic ketosis in patient with *HNF1B* mutation (c.826C>T, p.Arg276*). The occurrence of diabetic ketosis might be related to insufficient insulin secretion, which was different from type 1 diabetes in that diabetes-related autoantibodies were negative. While previous reports suggested that pancreatic hypoplasia was often the cause of insufficient insulin secretion in MODY5 patients (14), no overt pancreatic hypoplasia was presented in our patient, suggesting a possible non-pancreatic volume-dependent dysfunction. Notably, although the serum C-peptide levels were below the normal range, it did not reach deficiency. Insulin secretion was still preserved, and if this process persisted, MODY should be suggestive (15). Furthermore, there was no corresponding increase in postprandial C-peptide levels. A possible explanation was that *HNF1B* mutations might cause dysfunction of the GLUT-2 signaling pathway, resulting in insufficient glucose-stimulated insulin secretion (4). As the effect accumulated, the patient eventually developed diabetic ketosis. However, diabetic ketosis had not been reported in previous cases of *HNF1B* mutation (c.826C>T, p.Arg276*), and the possible reasons were as follows: First, there was obvious heterogeneity in MODY5 patients, and the clinical manifestations were different among different patients. Second, diabetic ketosis was a rare manifestation of MODY5 (16), which might not have received much attention in previous studies. Finally, there might be a cumulative effect in the development of diabetes and diabetic ketosis, which occurred when the disturbance of glucose metabolism caused by the mutation reached a certain level.

The patient presented with typical polycystic kidney manifestations, which was consistent with previous reports (13). Although studies have shown that patients with the mutation might have worse kidney damage than those with the gene deletion (13), kidney damage was not presented in this patient. Hypokalemia was also presented in this patient, and previous studies have shown that hypokalemia occurs in approximately half of adults with *HNF1B* mutations (17). *HNF1B* can directly regulate the transcription of genes related to renal K^+^ processing and can regulate the transcription of K^+^ channel proteins (12). Dysregulation of multiple transporters might contribute to hypokalemia. In addition, hypomagnesemia was also a common presentation in MODY5 patients, often due to renal magnesium loss (18). The degree of hypomagnesemia varied across age groups in patients with *HNF1B* mutations, and previous studies have shown that hypomagnesemia develops with increasing age (19). Therefore, although the patient’s current serum magnesium was within the normal range, regular monitoring of serum electrolytes was still necessary.

Insulin was the main recommended treatment agent for MODY5 patients, especially when *HNF1B* mutation (c.826C>T, p.Arg276*) occurred. Although some patients might be independent of insulin in the early stage (5), it should be noted that this mutation might lead to potential insulin secretion defects. Therefore, when MODY5 was suspected, pancreatic function should be evaluated and inappropriate drug should be avoided.

## Conclusion

In conclusion, we report a MODY5 patient with onset of diabetic ketosis in whom we identified a nonsense mutation at exon 4 of the *HNF1B* gene. Multiple renal cysts and youth-onset diabetes are common manifestations in patients with *HNF1B* mutations, and insufficient insulin secretion might be a potential cause of diabetic ketosis in MODY5. Our findings provide a new complement to the phenotype of MODY5 and bring more attention to patients with diabetic ketosis.

## Data availability statement

The original contributions presented in the study are included in the article/**Supplementary Material**. Further inquiries can be directed to the corresponding authors.

## Ethics statement

Written informed consent was obtained from the individual(s) for the publication of any potentially identifiable images or data included in this article.

## Author contributions

SG: Data extraction, Data analysis, Essay writing, and Paper submission. MY, WG and WC: Data extraction. JD and LL: Critical revision and Paper submission. All authors contributed to the article and approved the submitted version.

## Funding

This work was funded by the National Natural Science Foundation of China (82170847).

## Conflict of interest

The authors declare that the research was conducted in the absence of any commercial or financial relationships that could be construed as a potential conflict of interest.

## Publisher’s note

All claims expressed in this article are solely those of the authors and do not necessarily represent those of their affiliated organizations, or those of the publisher, the editors and the reviewers. Any product that may be evaluated in this article, or claim that may be made by its manufacturer, is not guaranteed or endorsed by the publisher.
