# Supplementary material for: Case Report: A case of HNF1B mutation patient with first presentation of diabetic ketosis
Source: Front Endocrinol (Lausanne). 2022 Aug 5;13:917819. doi: 10.3389/fendo.2022.917819 (PMC9388818; doi:10.3389/fendo.2022.917819)
Supplement: Supplementary file 1 [file Table_1.doc]

**Table S1 | *HNF1B* score in the case.**

| Characteristics | Item | Value | Case |
| --- | --- | --- | --- |
| Family history |  | 2 | - |
| Antenatal renal abnormalities | Uni/bilateral abnormality by renal echography | 2 | - |
| Kidneys and urinary tract |  |  |  |
| Left kidney | Hyperechogenicity | 4 | - |
|  | Renal cysts | 4 | 4 |
|  | Hypoplasia | 2 | - |
|  | Multicystic and dysplastic kidney | 2 | 2 |
|  | Urinary tract malformation | 1 | - |
|  | Solitary kidney | 1 | - |
| Right kidney | Hyperechogenicity | 4 | - |
|  | Renal cysts | 4 | 4 |
|  | Hypoplasia | 2 | - |
|  | Multicystic and dysplastic kidney | 2 | 2 |
|  | Urinary tract malformation | 1 | - |
|  | Solitary kidney | 1 | - |
| Electrolyte or uric acid disorders | Low serum Mg (＜ 0.7 mmol/l) | 2 | - |
|  | Low serum K (＜ 3.5 mmol/l) | 1 | 1 |
|  | Early-onset gout (＞ 30 years of age) | 2 | - |
| Pathological findings | Oligomeganephronia or glomerular cysts | 1 | - |
| Pancreas | MODY or hypoplasia of tail and neck of the pancreas or pancreatic exocrine insufficiency | 4 | - |
| Genital tract | Genital tract abnormality | 4 | - |
| Liver | Live test abnormalities of unknown origin | 2 | - |
